# Development of a Lizard-Inspired Robot for Mars Surface Exploration

**DOI:** 10.3390/biomimetics8010044

**Published:** 2023-01-18

**Authors:** Guangming Chen, Long Qiao, Zhenwen Zhou, Lutz Richter, Aihong Ji

**Affiliations:** 1 Lab of Locomotion Bioinspiration and Intelligent Robots, College of Mechanical and Electrical Engineering, Nanjing University of Aeronautics and Astronautics, Nanjing 210016, China; 2 Large Space Structures GmbH, Hauptstr. 1e, D-85386 Eching, Germany

**Keywords:** Mars robot, flexible spine, foot trajectory, planetary rover, space exploration

## Abstract

Exploring Mars is beneficial to increasing our knowledge, understanding the possibility of ancient microbial life there, and discovering new resources beyond the Earth to prepare for future human missions to Mars. To assist ambitious uncrewed missions to Mars, specific types of planetary rovers have been developed for performing tasks on Mars’ surface. Due to the fact that the surface is composed of granular soils and rocks of various sizes, contemporary rovers can have difficulties in moving on soft soils and climbing over rocks. To overcome such difficulties, this research develops a quadruped creeping robot inspired by the locomotion characteristics of the desert lizard. This biomimetic robot features a flexible spine, which allows swinging movements during locomotion. The leg structure utilizes a four-linkage mechanism, which ensures a steady lifting motion. The foot consists of an active ankle and a round pad with four flexible toes that are effective in grasping soils and rocks. To determine robot motions, kinematic models relating to foot, leg, and spine are established. Moreover, the coordinated motions between the trunk spine and leg are numerically verified. In addition, the mobility on granular soils and rocky surface are experimentally demonstrated, which can imply that this biomimetic robot is suitable for Mars surface terrains.

## 1. Introduction

Space exploration is important for humankind to contribute to comparative planetology and discovering new resources out of the Earth, and future human missions to bodies outside the Earth are being prepared [1,2]. To achieve these goals, a series of activities have been ongoing by spacefaring actors. After the recent successes in Mars exploration by NASA with several generations of six-wheeled vehicles of different size ranges, the United States is currently developing the ‘VIPER’ four-wheeled rover with active suspension for exploration of the polar regions of the Moon [3]. European nations are ready to send the ‘Exomars’ Rover to Mars which will perform subsurface drilling [4]. China has operated ‘YuTu’ rovers on the moon and ‘ZhuRong’ on Mars [5]. The United Arab Emirates is sending the ‘Rashid’ rover to the moon [6]. Mars surface exploration currently mainly comprises investigations of the surface geology and geomorphology [7], study of mineralogy and chemistry of key material units at the landing sites through sample acquisition and analysis [8], and instrument deployment. In the future, infrastructure construction in support of human exploration is expected to constitute another group of tasks. In support of such missions, planetary surface rovers are indispensable. The Mars surface is characterized by soil-like material and rocks of different sizes [9]. In the attempt to adapt to the Mars surface terrains, several types of planetary rovers have been proposed.

Contemporarily, wheeled structures are commonly used for planetary surface rovers. The advantages of such rovers are high agility, high speed, and a simple control system. Nonetheless, wheeled rovers may encounter severe sinkage and embedding in very soft weak soils [10]. The ‘Zhurong’ rover possesses the strategy of escaping from sinkage using the active control suspension system for lifting of wheels [11]. Nonetheless, it requires large torque to operate an embedded wheel. Leg-wheeled robots are based on the structures and mechanism of animal legs, so that they can promote higher maneuverability in cornering and avoiding obstacles (e.g., the NASA’s ‘Athlete’ [12], and ‘SherpaTT’ [13]). Nevertheless, the wheels can suffer from high slip for the soft soils [14]. Another type of the legged rover, Spacebok [15], has a simple foot structure and better adaptability on soft soil surface. However, it is unqualified to adapt to non-flat terrains. The rock-climbing robots which adopt grippers on the foot toes, such as Lemur 3 [16] and Nagaoka’s robot [17], are capable of climbing on vertical, and inverted rock surfaces. Nevertheless, their trunks are made of rigid structures, resulting in a poorer balancing capability and walking speed compared to real quadruped animals.

Among the legged rovers, the quadrupedal robots are distinguished from other legged robots in producing effective motions with relatively uncomplicated structure [18]. Our previous model of a desert Chameleon-inspired robot has successfully demonstrated the potential of adapting to Mars surface terrains [19]. Moreover, the flexible spine enables larger strides compared to rigid trunks. However, the stand–walking feature of the robot structure results in higher center of mass, causing unstable motion as walking speed increases. Furthermore, the lift height of each leg is not sufficient, which reduces the ground clearance in traversing rocks. For increasing the adaptability for Mars’ surface terrains, an innovative structure of the quadruped robot based on other inspiration can be used.

Toward future applications of Mars surface geomorphology exploration and detection with loaded scientific instruments, the work described here contributes a biomimetic quadruped robot based on the structure and mechanism of a creeping animal desert lizard which moves efficiently on both granular soils and rocky surface. It can produce a steadier walking motion due to a lower center of mass and updated joint structures. To ensure coordinated movements among the foot, leg, and spine, the combined kinematics model are established based on their simultaneous motions during walking. Finally, the mobility on granular soils and rocks in analogy to the Mars surface are experimentally demonstrated. The results infer that the biomimetic robot is promotable for adapting to Mars surface terrains.

## 2. Biomimetic Structure Design

To propose a bionic creeping quadruped robot based on a desert lizard, the locomotion characteristics are illustrated next. Referring to biological characteristics, the biomimetic structure design of flexible spine, leg, and multi-toe foot is elaborated. Finally, the biomimetic structure of a lizard-inspired robot is presented.

### 2.1. Biological Characteristics

**Figure 1:**
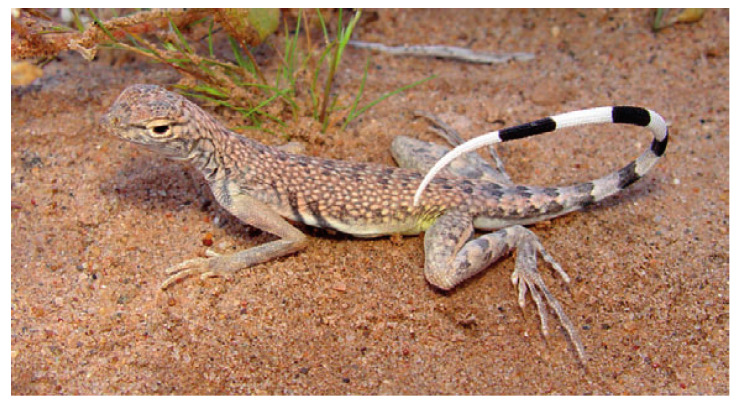
A desert lizard (Photo credit: Thomas C. Brennan) [21].

Figure 1 shows a desert lizard that can efficiently move on granular sands and rock surface. The efficient movements on granular sand and rocky surfaces are mainly ascribed to flexible spine, creeping legs, and graspable toes. The spine contains a series of active flexible joints. Each leg can be decomposed of swing thigh and shank. During walking, the shanks of the leg swing in synchronization with motions of the flexible spine [20]. For adapting to the soft desert surface, they use large contact pads and multiple toes [21]. The footpad can increase the contact area with respect to granular sands to prevent subsidence. The grasping toes can solidify the loose sands and thus achieve steady reaction forces.

### 2.2. Biomimetic Structure

**Figure 2:**
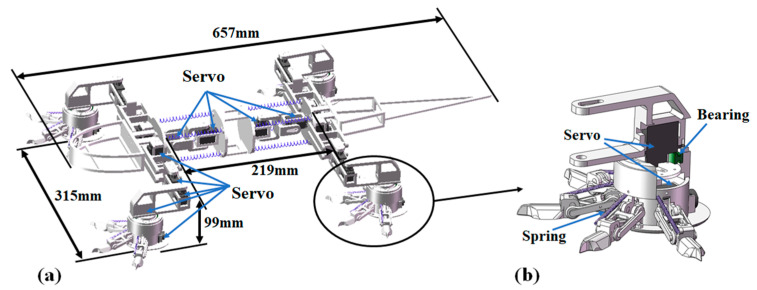
Desert lizard-inspired robot: (**a**) biomimetic structure and (**b**) break out section of ankle.

To design a flexible spine as that of the lizard, four servos are used to generate two left–right motions and two up–down motions for the symmetric biomimetic flexible spine [22], as shown in Figure 2a. These four gears are connected by different brackets. Eight springs are used to connect the hip joints and the middle bracket of the spine, which increase the loading capability and reduce vibrations. For achieving a lizard-like creeping motion, each leg adopts two hinges and each require one gear to produce a swinging movement. The hip joint between the spine and leg is composed of two servos and a four-linkage mechanism which can promote a stable lift. A servo is also used to join the thin and the shank.

The thigh and footpad are connected by an active rotational ankle that is composed of a servo and a bearing, as given in Figure 2b. A servo is placed between the footpad and the bearing. It connects these four tension springs by a rope and generates active rotational motions to adjust to contact positions of foot toes. The foot pad connects four flexible toes. Each toe consists of two hinges and a claw at the tip. For each toe, a tension spring is used to connect the pad and the claw [23]. A servo is embedded inside the round pad, and when the rope is tensioned by the servo, the four toes grasp downward.

## 3. Kinematics Modelling

To evaluate the performances of the biomimetic robot, kinematics studies for the spine, leg, and foot are conducted. Moreover, the foot trajectory for forward motion is elaborated and the workspace of the foot is estimated. Finally, the gait planning for robot forward walking is determined.

### 3.1. Leg Motion Determination

**Figure 3:**
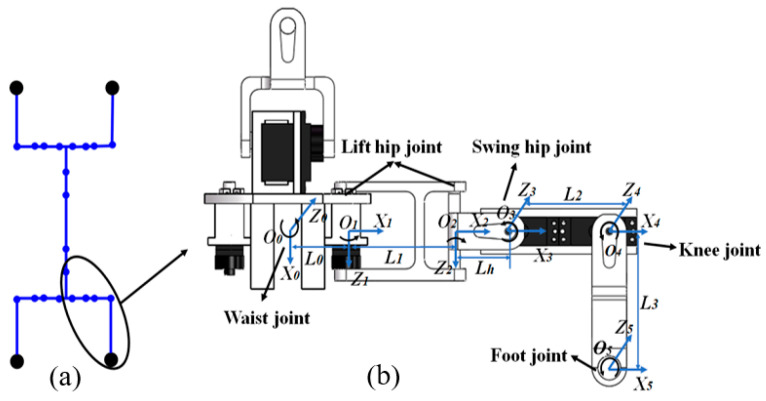
Leg motion analysis: (**a**) joints of the robot diagram and (**b**) coordination systems.

According to the biomimetic structure, the spine has four joints, and each leg has four joints. All the joints of the robot are denoted in the schematic robot diagram of Figure 3a. As the robot moves, the ends of the spine rotate with respect to the central line. Figure 3b illustrates the coordinate systems for spine end and whole leg, in which the end of the spine is the base coordinate system. The spine end is connected with the hip joint via a four-linkage structure. It can be rotated when driven by a motor, which produces an active degree of freedom (*O*_1_). The other end utilizes a connecting rod (*O*_2_) which is passively rotated in correspondence to the steady up–down motion of the leg. Therefore, the angles of active degrees of freedom (*O*_1_) and passive degrees of freedom (*O*_1_) are of the same magnitude and in opposite directions. A video of the hip joint motion is provided to illustrate the hip joint motions (Supplementary File). The knee and foot, respectively, have the active freedoms *O*_3_ and *O*_4_.

Based on the coordinate system shown in Figure 3, the D–H parameters can be obtained as given in Table 1. By transformations of the pose matrix, the positions of each joint can be resolved as the claw [24].

To better control the movement of the robot, the position of each joint corresponding to the robot foot trajectory must be predetermined. Assuming that the spine swings at a constant speed, and denoting $\theta_{max}$ the maximum rotational angle of the left trunk end, the swinging angle $\theta_{0}$ as a function of time *t* can be expressed by

(1) $$\theta_{0}=\theta_{max}*sin\left(t*\frac{\pi }{4}\right)$$

For conveniently solving the freedoms of the leg, the hip joint (*O*_1_) is used as base coordinate system. The trajectory that was expressed by the waist joint (*O*_0_), is transformed to estimate positions of the three active joints (*O*_1_, *O*_3_, and *O*_4_). The foot position that was established as referring to the waist joint (*O*_0_) can be expressed by hip joint (*O*_1_), which is

(2) P01=T−110∗P0=P0,X1P0,Y1P0,Z1=PX0∗cosθ0+PY0∗sinθ0−L0PZ0PX0∗sinθ0−PY0∗cosθ01

Using the hip joint (*O*_1_) as the base coordinate system, the position of the foot joint (*O*_4_) can be obtained,

(3) P1=T51∗0001=L1∗cosθ1+L2∗cosθ3+L3∗cosθ3+θ4L1∗sinθ1−L2∗sinθ3−L3∗sinθ3+θ41

Using inverse dynamics, the trajectories of three active joints (*O*_1_, *O*_3_, and *O*_4_) can be obtained based on the hip joint (*O*_1_) [25], which is given by Equation (4).

(4) θ1=arcsinPY1/L1θ2=−arcsinPY1/L1θ3=arccos1PZ2−L32∗sinθ42L3∗sinθ4∗PX1−Lh−L1∗cosθ1−PZ1∗L2+L3∗cosθ4θ4=arccosPX1−Lh−L1∗cosθ12+1PZ2−L22+L322∗L2∗L3

Assuming the waist joint (*O*_0_) as the base coordinate system and substituting joint functions PX1, PY1, and PZ1 in $\theta_{1}$, $\theta_{2}$, $\theta_{3}$, and $\theta_{4}$ with position functions P0,X1, P0,Y1, and P0,Z1 of Equation (2), the motion function of each joint can be solved in correspondence to the designated foot trajectory.

To promote a smooth up–down motion of the foot with respect to the irregular terrains of the Mars surface, the composite non-impact curve is generally recommended to determine the foot trajectory [26]. Moreover, this type of trajectory can be adjusted by changing the step length and step height in accordance with the robot motion mechanism. The designated non-impact composite curve trajectory is given by Equations (5)–(7) [27],

(5) $$P_{X}\left(t\right)=\left\{\begin{array}{c} s*\left(\frac{t}{T}-\frac{sin\left(2*\pi *t/T\right)}{2*\pi }\right)\;\left(\frac{t}{T}\geq 0\&\frac{t}{T}\leq \frac{1}{2}\right)\\ P_{X}\left(1-\frac{t}{T}\right)\;\left(\frac{1}{2}\geq \frac{t}{T}\&\frac{t}{T}\leq 1\right) \end{array}\right.$$

(6) $$P_{Y}=\left(L_{0}+L_{1}+L_{2}\right)\;\left(t\geq 0\&t\leq T\right)$$

(7) $$P_{Z}\left(t\right)=\left\{\begin{array}{c} 2*h*\left(\frac{t/T}{1-k}-\frac{sin\left(4*\pi *\frac{t/T}{1-k}\right)}{4*\pi }\right)\;\left(\frac{t}{T}\geq 0\&\frac{t}{T}\leq \frac{1-k}{2}\right)\\ h\;\left(\frac{t}{T}\geq \frac{1-k}{2}\&\frac{t}{T}\leq \frac{1+k}{2}\right)\\ P_{Z}\left(1-\frac{t}{T}\right)\;\left(\frac{t}{T}\geq \frac{1+k}{2}\&\frac{t}{T}\leq 1\right) \end{array}\right.$$

In which *s* represents stride length, *h* represents leg lift height, *u* is the time variable, *T* is the period of movement, and *k* is the time parameter of forward movement. When *t*/*T* = 1, the swing phase of the foot trajectory is completed.

### 3.2. Work Space Estimation

**Figure 4:**
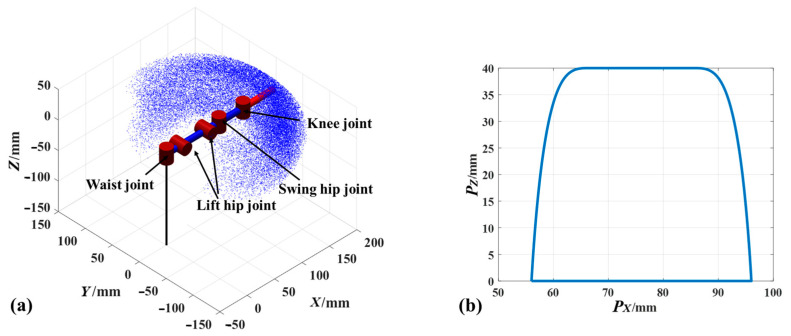
Movement of leg and foot: (**a**) workspace of leg and (**b**) wobble phase of foot.

To estimate movement space of the legs of the robot, the robot toolbox [28] can be used. The three lengths, namely $L_{1}$, $L_{2}$, and $L_{3}$ of the robot leg are 41 mm, 74 mm, and 150 mm, respectively. For the quadruped robot with a flexible spine, the foot movement space is affected by the swinging space of the spine. According to the applied robot leg lengths, the predicted motion ranges of the legs swinging with a flexible spine on the X, Y, and Z axis are 184 mm, 250 mm, and 80 mm, respectively, as shown in Figure 4. For a given robot foot step size of 40 mm, height of 40 mm, and swing phase of 2 s, the swing phase trajectory can be obtained (Figure 4b) by using Equations (5)–(7).

### 3.3. Gait Planning

**Figure 5:**
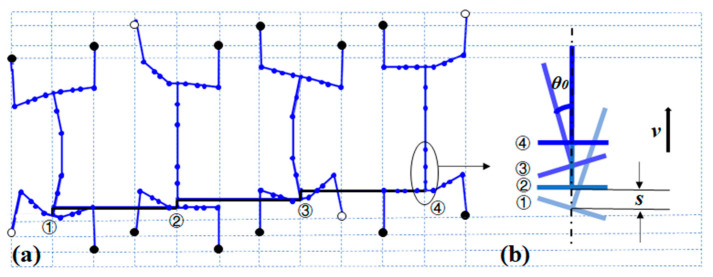
Gait planning: (**a**) four steps of the gait and (**b**) illustration of variations in the trunk end.

The previous section analyzed the forward and inverse kinematics of part of the spine to the legs of the robot, which have been solved. In order to enable the whole robot to stably move forward, effective gait planning is crucial [27]. Based on the crawling lizard gait at the open space, a triangular gait is implemented. Figure 5 illustrates the gait plan of the robot in a straightforward motion. At step ①, the right front and left hind legs subsequently lift forward, and meanwhile the spine bends right. Next, the left hind and left front feet move forward as the spine returns to the straight line (Step ②). Then, the right hind leg moves forward and the spine bends left (Step ③). Finally, the robot returns to the initial position while the right front foot moves forward (Step ④). For the four steps, the positions of the same end of the spine are zoomed-in Figure 5b. The advancing distance between each step is set as *s. v* is the forward velocity and *θ0* is the swinging angle about central line.

During straight walking, the velocity of the spine is maintained as constant. The velocity of the front spine consists of two components, i.e., the swinging and the forward velocity of the spine. Therefore, the movement of each leg must be calculated separately. The left hind leg moves forward when the spine bends to the right. Assuming that the position of the entire spine remains unchanged, the wobble phase of the left hind foot can be regarded as the utilized non-impact motion, and the support phase can be a forward walking motion. Thus, the motion curve of the foot in the forward direction ($P_{X}$) is modified as

(8) $$P_{X}\left(t\right)=\left\{\begin{array}{c} L_{3}-s*\left(\frac{t}{T}-\frac{sin\left(2*\pi *t/T\right)}{2*\pi }\right)\;\left(\frac{t}{T}\geq 0\&\frac{t}{T}\leq \frac{1}{2}\right)\\ L_{3}-s+s*\frac{t-2}{6}\;\left(\frac{t}{T}\geq \frac{1}{2}\&\frac{t}{T}\leq 4\right) \end{array}\right.$$

## 4. Verification by Simulation

To verify the theoretical results of robot motions, a simulation approach using Adams software can be used [25,27]. This section models the leg motion according to the adopted theoretical results of kinematics studies. Moreover, the walking characteristics by the prescribed gait are predicted. In addition, the grasping motion of the foot toes is simulated.

### 4.1. Leg Motion

**Figure 6:**
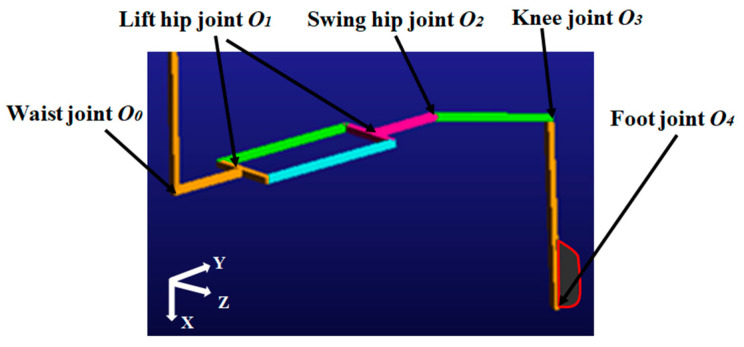
Foot trajectory simulation.

To efficiently verify the results of the leg motion by simulation, the structure of the leg and spine end (Figure 3b) can be simplified, which is shown in Figure 6. Different colors represent different parts of the robot. The corresponding rotation pairs and the drives for the joints between each part are applied. The motion curve of Equation (8) is implemented in simulations and the corresponding motion trajectory of the foot is obtained as shown by the red curve.

**Figure 7:**
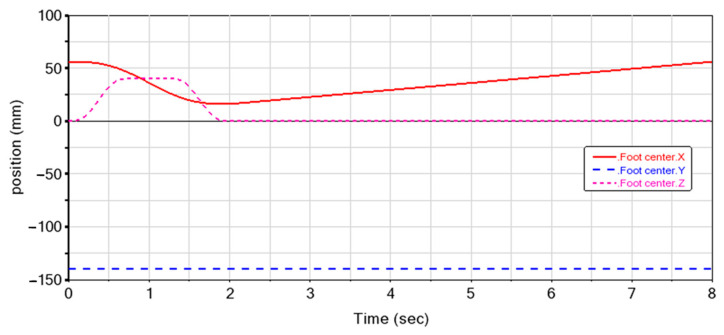
Variation of foot positions in three axes in simulations.

Figure 7 shows the obtained trajectory for one cycle period. On the X axis, the foot center decreases from 56 mm to 16 mm after 2 s, and then linearly increases to 56 mm. The magnitude is maintained at 140 mm on the Y axis, which is consistent with the set foot movement trajectory. The magnitude of the motion curve on the Z axis increases to 40 mm after 0.75 s, and returns to zero at 2 s. The variations in X and Z axes are consistent with the prescribed motion as shown in Figure 4b. Thus, the correctness of the theoretical analysis is verified.

### 4.2. Gait Simulation

The gait simulation involves material parameters and contact parameters between the foot and ground surface. The foot structures are printed using resin materials. The spring material is manganese steel. To estimate the effectiveness of theoretical gait planning, the ground surface can be modelled as concrete. The contact parameters are referred to the Adams software manual [29]. The simulation parameters are given in Table 2.

**Figure 8:**
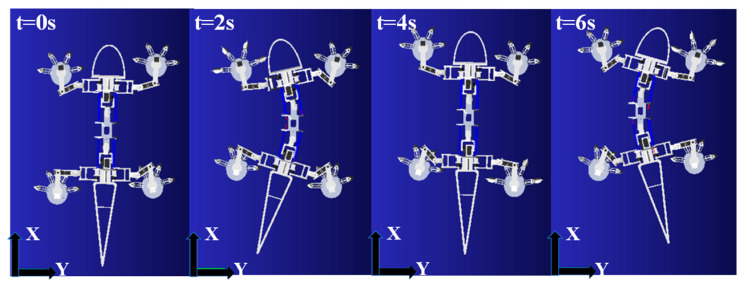
Gait simulation of robot forward walking.

Using the parameters in Table 2 and the predetermined gait (Figure 5), the robot motions for one cycle are simulated as shown in Figure 8. It demonstrates that the legs and spine of the robot can cooperate with each other and move forward stably. The period of a full cycle of the robot movement is 8 s as prescribed in theory. Therefore, the simulation results verify that the theoretical gait plan can be used for the robot to effectively perform forward walking.

**Figure 9:**
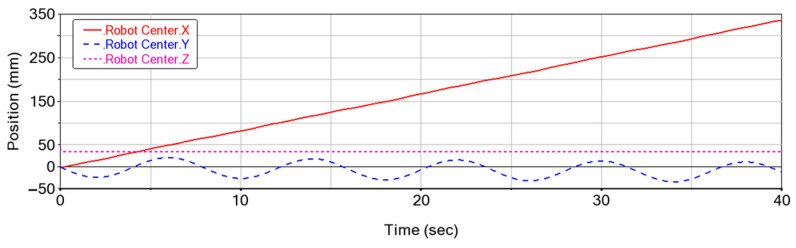
Variations in the center of mass for five walking cycles.

To assess the stability of the forward motion, the variations in the robot’s center of mass for five cycles are shown in Figure 9. It can be seen that the robot generates periodic changes when it advances. In each cycle, the increment of the robot in the forward direction (X axis) is 66.7 mm, which is close to the estimations using Equation (8). The change to spine swinging motions (Y axis) is 45.2 mm. The deviation in the Y axis direction is 2.04 mm, which is caused by the slippery aspect of the foot. The position in the vertical direction (Z axis) is maintained as constant, which is because no up and down movements of the spine were implemented. The average moving speed is calculated at 8.34 mm/s, which can be also estimated by Equation (8).

### 4.3. Grasping Simulation

**Figure 10:**
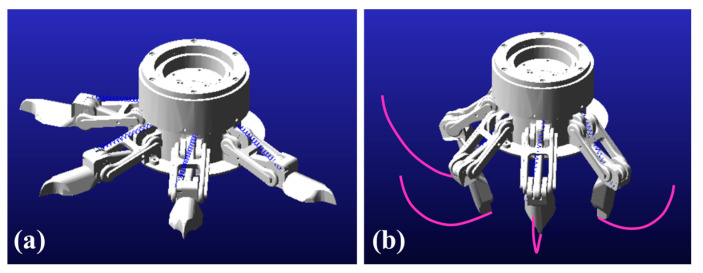
Grasping behavior simulation: (**a**) retrieving state and (**b**) grasping state.

The foot toes initially rest in a relaxed open state, and will bend downward by the driven torques. Figure 10 shows the simulation results, in which the red curves are the trajectories for the bending motion. The maximum rotation angle (*θ*) of the toes is about 120°, which is expected based on the structure and geometric sizes. However, in practice, the bending angles are highly affected by the stiffness of the contact surface.

## 5. Experimental Tests

In accordance with the dimensions of the structure model in Figure 2, a prototype of the biomimetic robot was fabricated. Using a simulated test bed, the grasping behaviors of the foot toes with respect to granular soils were evaluated. Moreover, the mobility on the surface analogy to the Mars terrains were tested.

### 5.1. Fabrication

**Figure 11:**
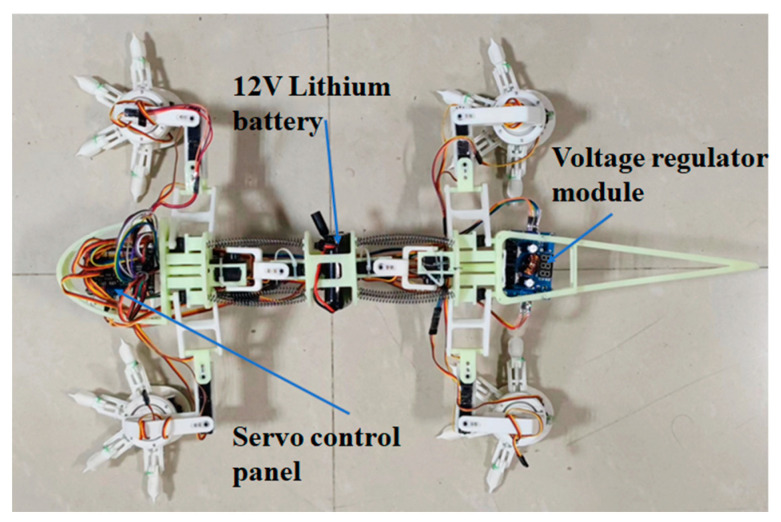
The fabricated prototype of the lizard-inspired quadruped robot.

The components for foot, leg, and spine were fabricated using resin materials by 3D printing. A 32-way servo control panel is used to send signals to servos to enable component motions. A lithium battery (12V) is used to supply electricity to the control panel. Meanwhile, a voltage regulator module (XL4016E1DC-DC) is used to adjust central interface and power supply. The type of the 24 servos are PTK 7455MG-D12g which are commercially purchased from the company of *Mayatech*. Using high accuracy potentiometers, the deviations of rotation angles from the servos are transferred to the control panel, where the signals are processed and sent back to servos to adjust the positions and voltages. The servo adopts asynchronous serial bus communication, so that the port can send and receive data through different wires. In this manner, simple and long-distance data transfer can be achieved using less complicated wire configuration [30]. The fabricated biomimetic robot prototype is shown in Figure 11.

### 5.2. Mobility Test

**Figure 12:**
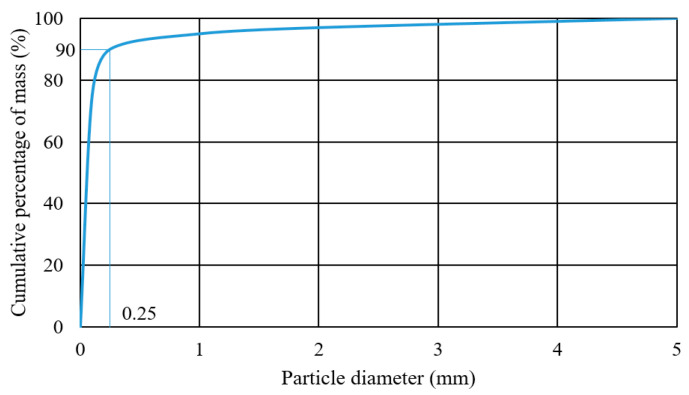
Particle size distribution of the soil used in the testbed.

**Figure 13:**
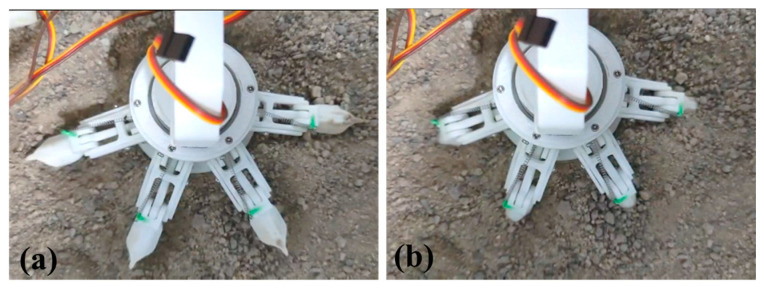
Grasping tests: (**a**) retrieving state and (**b**) grasping state.

Referring to the Martian soil properties [31,32], a testbed was made for mobility test. Figure 12 presents the particle size distribution, which shows that the majority mass (75%) are the particles whose sizes are smaller than 0.1mm. The bulk grain density is around 2737 kg/m^3^. Figure 13 shows the grasping tests of foot toes with respect to the simulated surface. When the motor drives the rope, the toes bend down into soils, and a grasping angle of higher than 90° is obtained.

**Figure 14:**
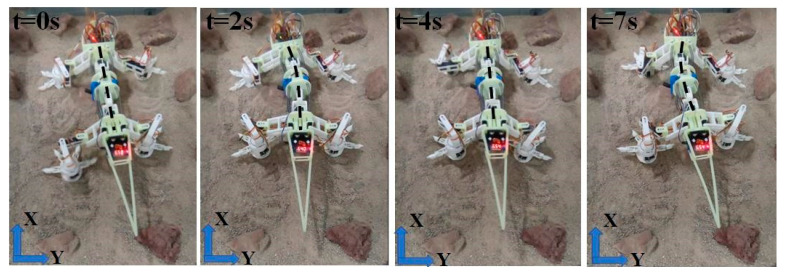
Moving on simulated Mars surface terrains.

In order to model the rocky environment as that of the Mars surface, a selection of rocks with sizes 60–120 mm were embedded in the granular materials [33]. Figure 14 shows the four postures of the robot when walking by a straight line on the simulated surface. It is observed that its spine swings in cooperation with the foot movement. The bending motions are weaker than the simulation results. The reason for this is that the support springs on the spine counteract some moments of the servos. Consequently, the average speed of the robot is also lower (approximately 5.1 mm/s) than simulations. Nonetheless, the experiments demonstrated that this robot could maintain forward motion on the rocky surface by the implemented theoretical gait plan.

**Figure 15:**
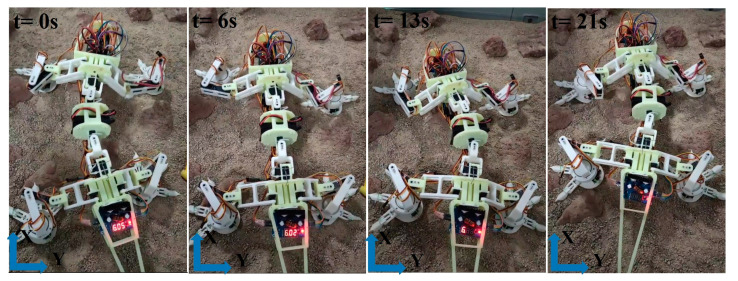
Climbing over a rock on the simulated Mars surface terrains.

To further demonstrate that the robot is suitable for rocky environments, external tests were conducted and are shown in Figure 15. It can be identified that the left hind leg of the robot can climb across a rock, indicating that this improved robot model has better performance compared with the previous work [19].

## 6. Discussion

To explore geomorphology and conduct surface exploration on Mars with scientific instruments carried by a robot, this work evaluates a biomimetic creeping robot inspired by the desert lizard animal [34]. Its mobility with respect to soft granular soils and rocks as those of the Mars surface were assessed, which demonstrates that this robot is adaptive to both granular and rocky terrains, as opposed to the wheeled rovers [11] and legged rovers [12,13]. The flexible spine promotes larger swinging ranges and thus a larger stride compared to rigid spine robots [17]. Compared to Lemur [16], fewer and more flexible toes are used, which reduce the structure complexity and is beneficial to efficient control. Moreover, the design of creeping legs enables a lower center mass and higher stability which is more advantageous compared to a stand–walking robot [19]. Specifically, the leg adopts a four-linkage structure which ensures steady lift of the foot. Therefore, the structure for this robot can perform a move stable movement than our previous model [19].

To achieve coordinated movement between the foot and spine, the trajectory was formulated by integrating motions of the foot, leg, and spine. The foot trajectory adopts a composite curve such that excessive impacts when contacting the ground are avoided. The foot motion can be regulated by adjusting step length or height as long as the robot motion mechanism is retained. Moreover, this trajectory has certain adaptability for climbing different slopes, which promotes higher adaptability to the Mars surface environment [27]. For a faster forward speed, the weight distribution of the spine can be optimized by the structure design. To further improve the walking stability for irregular surfaces, gait planning, by considering lizard locomotion on a complex environment, may be studied [35].

The regolith and terrains of planetary surfaces vary for different locations. Correspondingly, the experimental mobility tests on such terrains is essential to also be tested at lower gravity levels [9]. Furthermore, it is important to perform the resilient locomotion of the robot, which would be beneficial to improving the design and control, and thus the adaptability to Mars surface terrains [36]. On the other hand, numerical simulations based on MBD-DEM [37] can be used to model the walking characteristics. A full sealing of the robot drive mechanism and hinges is necessary to protect against particle ingress from the Mars soil and airborne dust [38]. Considering launch and landing mechanical mission loads, radiation, and thermal effects, the rigid part of the robot structure for a flight model design would be replaced by titanium alloys, and the soft part such as that for electronic components would adopt fiber reinforced composite materials. To achieve adaptive control, the foot reaction force may be measured and the control strategy would then be adjusted [39]. Implementation of autonomous control of self-adapting to different terrains using machine learning is already undergoing [40]. In addition, the requirement for the continuous power supply in this quadruped robot is to be accounted for in the next version [41].

## 7. Conclusions

To adapt to the Mars surface terrains, this research proposed a biomimetic quadruped robot based on the structure and mechanism of a desert lizard. Kinematic models for coordinated movements among the foot, leg, and spine were established. To verify these models, the leg motion and straight walking abilities based on gait planning were analytically predicted. Furthermore, experimental tests demonstrated that the biomimetic robot is suitable for granular soils and rocky surfaces, which is of high potential for walking on the Mars surface terrains. Thus, this work progresses the development of Mars robots for surface exploration.

## Figures and Tables

**Table 1:** D–H parameter table.

Link	Torsional Angle *α*	Link Length *L*	Joint Angle $\theta_{n}$	Joint Distance $d_{n}$
1	90°	*L*_0_	$\theta_{0}$	0
2	0°	*L*_1_	$\theta_{1}$	0
3	−90°	*L_h_*	$\theta_{2}$	0
4	0°	*L*_2_	$\theta_{3}$	0
5	0°	*L*_3_	$\theta_{4}$	0

**Table 2:** Simulation parameters.

Categories	Parameters	Values
Materials	Steel density	7801 Kg/m^3^
	Steel Young’s Modulus	2.07 GPa
	Steeel poisson ratio	0.29
	Spring stiffness	4.6 N·mm/°
	Concrete	2000 Kg/m^3^
Contact	Stiffness	4855 N/mm
	Damping	80 N s/mm
	Penetration depth	0.1 mm
	Static coefficient	0.7
	Dynamic coefficient	0.57
	Static transition velocity	0.1 mm/s
	Friction transition velocity	10 mm/s

## Data Availability

The datasets generated during and/or analyzed during the current study are available from the corresponding author on reasonable request.

## Supplementary Materials

The following supporting information can be downloaded at https://www.mdpi.com/article/10.3390/biomimetics8010044/s1.
